# Structural Investigation of Diclofenac Binding to Ovine, Caprine, and Leporine Serum Albumins

**DOI:** 10.3390/ijms24021534

**Published:** 2023-01-12

**Authors:** Julita A. Talaj, Kamil Zielinski, Anna Bujacz

**Affiliations:** 1 Department of Molecular Biology of Cancer, Medical University of Lodz, 6/8 Mazowiecka Street, 92-215 Lodz, Poland; 2 Department of Hematology, Comprehensive Cancer Center and Traumatology, Copernicus Memorial Hospital, Pabianicka Street 62, 93-513 Lodz, Poland; 3 Institute of Molecular and Industrial Biotechnology, Lodz University of Technology, B. Stefanowskiego 2/22, 90-537 Lodz, Poland

**Keywords:** ovine serum albumin, caprine serum albumin, leporine serum albumin, diclofenac, non-steroidal anti-inflammatory drug, crystal structure

## Abstract

Free drug concentration in the blood sera is crucial for its appropriate activity. Serum albumin, the universal blood carrier protein, is responsible for transporting drugs and releasing them into the bloodstream. Therefore, a drug’s binding to SA is especially important for its bioavailability and it is a key problem in the drug design process. In this paper, we present crystal structures of three animal serum albumin complexes: ovine, caprine, and leporine, with diclofenac, a popular non-steroidal anti-inflammatory drug that is used in therapy of chronic and acute pain. Details of diclofenac binding mode by the presented serum albumins are compared with analogous complexes of human and equine serum albumins. The analysis of the occupied binding pockets in crystal structures of the investigated serum albumins from different mammals shows that they have two common and a number of unique diclofenac binding sites. The most intriguing is the fact that the albumins from the described species are able to bind different numbers of molecules of this popular anti-inflammatory drug, but none of the binding sites overlap with ones in the human serum albumin.

## 1. Introduction

Drug pharmacokinetics is one of the most important issues in pharmacological therapy. The effect of a drug on a patient is dependent on its concentration in the plasma at the target site where the drug molecules are to be bound to specific receptors. However, the majority of pharmaceutics are poorly soluble in physiological conditions. They may be dangerous to patients due to potential drug overactivity that is caused by high drug concentration or toxic effects [1]. After administration of the drug into a patient’s body its molecules are bound by plasma proteins, mostly by serum albumin, and that influences the drug’s overall biological activity, metabolism, distribution, and processes of elimination and excretion [2].

Serum albumin (SA) is the most significant transport protein of vertebrates; it constitutes up to 60% of all plasma proteins. It is also well known for its outstandingly wide binding properties, which are an effect of the flexibility of its helical structure. SA consists of three loose domains, that are almost structurally identical and mostly helical. The whole structure is maintained by 17 disulfide bridges, which ensure durability of the overall protein fold and simultaneously allow its flexibility [3,4]. During decades of research on HSA, numerous binding sites have been identified and they were classified as seven sites for binding long and medium chain fatty acids (FA1-FA7) [5], and two main sites for binding small organic compounds, named drug sites I and II (DSI, DSII) [6]. However, albumins have additional binding sites for short chain fatty acids, hormones, metabolites, toxins, metal ions, and medicaments. SA binds the majority of active substances, therefore, investigations of its interactions with drugs are very important [7,8,9]. Any potential drug or inhibitor should ensure the appropriate affinity to the target protein or enzyme, as well as to the transporting protein (albumin). The latter one is a key problem in the drug design, as it should ensure a proper drug release [2]. This research is also connected to the cross activity effect that occurs when the presence of one compound influences the binding of another one. It is dependent on the affinity of binding of both substances and it was perceived as a reason for numerous side effects that are connected to long-term pharmacotherapy. Recently, this effect is considered as an alternative therapy, allowing the minimization of drug dose by using another compound, having affinity to the same SA binding pocket, as a trigger of drug release [10,11,12,13]. For that purpose, it is necessary to understand the exact drug binding properties to SA. For most drugs, the primary binding site is maintained among albumins of various mammals, but a secondary drug location is often typical only for an albumin from a particular organism.

Diclofenac (DIC, 2-(2,6-dichloranilino)phenylacetic acid) is a worldwide known non-steroidal anti-inflammatory drug that is used in various diseases that are associated with severe muscle or bone pain, such as rheumatoid arthritis, osteoarthritis, or soft tissue injuries. It is also tested as an active compound in Alzheimer disease therapy [14,15]. This drug, belonging to phenylacetic acid derivatives group, prevents inflammation state development by blocking cyclo-oxygenase (COX) and phospholipase A2, suppressing the production of proinflammatory compounds [16,17,18,19]. The DIC application requires comprehensive investigations on its activity, effects of cross-activity, and pharmacokinetics. Various affinity constants of binding DIC in SA, depending on methodology, as well as different predictions about its localization, can be found in the literature [20,21,22,23,24,25,26]. The affinity constant of diclofenac to HSA, using isothermal titration calorimetry (ITC) in a temperature range from 20 to 40 °C, for 20 °C was *K*_a_ = (2.31 ± 1.17) × 10^4^ M^−1^, increased in 30 °C to *K*_a_ = (7.29 ± 4.36) × 10^4^ M^−1^ and in higher temperatures again decreased to *K*_a_ = (2.92 ± 0.32) × 10^4^ M^−1^. In BSA, a similar correlation of diclofenac binding affinity depending on temperature is observed, but the highest affinity *K*_a_ = (5.11 ± 1.22) × 10^4^ M^−1^ was at 35 °C [20]. The binding constant for diclofenac/HSA measured by fluorescence spectroscopy in the pH range of 6.0–8.0 shows that with a growing pH, diclofenac affinity to HSA was decreasing from 4.25 × 10^4^ M^−1^ to 1.70 × 10^4^ M^−1^ [24]. Determined diclofenac binding constants to BSA, by affinity capillary electrophoresis (ACE), indicates high and low diclofenac affinity binding sites: *K*_a_ = (1.9 ± 0.2) × 10^5^ M^−1^ and *K*_a_ = (2.4 ± 0.2) × 10^4^ M^−1^, respectively [26]. There are two association constants for DIC bound to ESA that were determined (*K*_a1_ = 5.13 × 10^6^ M^−1^, *K*_a2_ = 3.90 × 10^4^ M^−1^) [27]. Generally, authors agree on the existence of two main diclofenac binding sites in HSA, BSA and ESA, but only for ESA the exact diclofenac binding places, correlated with higher and lower affinity constants, are confirmed by crystal structures.

The structures of equine serum albumin (ESA) and human serum albumin (HSA) in complexes with diclofenac available in PDB under accession codes 4ZBQ and 4Z69, respectively [27,28], show different drug locations in ESA and HSA. However, it is still difficult to conclude a common diclofenac binding mode. Structural studies that are described in this paper regarding interactions of DIC in binding pockets of three serum albumins from different species, sheep (OSA), goat (CSA), and rabbit (LSA), answers the question about a common diclofenac binding place in these mammalian SAs. This knowledge makes a great contribution to understanding the mechanism of this drug’s distribution and its bioavailability. The use of this drug in animals is controversial due to its toxicity. DIC can kill scavenging birds that eat dead animals, and for this reason, it has been forbidden for veterinary use in many countries [29,30,31].

## 2. Results and Discussion

In this research paper, we describe three structures of serum albumin complexes with diclofenac (DIC): OSA-DIC, CSA-DIC, and LSA-DIC. Serum albumins of different species are structurally very similar; their overall folding is highly conserved during the evolutionary process, although their amino acid sequences differ. The sequence identity between two of the investigated albumins (OSA and CSA) is very high and it is equal to 98.5%, but the sequence similarity of OSA and CSA to LSA is much lower and it is equal to 72.4%. Despite this relatively high sequence identity between OSA and CSA, differences in ligand binding modes are visible, and they are more noticeable for the LSA-DIC complex. OSA and CSA are equipped with an additional tryptophan residue at position 134, which is characteristic for serum albumins of ruminants. There is also an insertion in the LSA sequence, which causes a shift in amino acid numbering in relation to OSA and CSA.

Diclofenac binding by investigated serum albumins causes some slight changes in their spatial structures with respect to unliganded albumins. In CSA-DIC, a significant change in the conformation of the loop (500–508) surrounding the FA5 pocket is observed. In OSA-DIC, the crystal structure binding diclofenac molecule induced changes in the conformation of amino acids side chains in the surroundings and, as a consequence, slightly shifted two helices in the Ib subdomain. In the LSA-DIC complex, some changes in the IIIb domain are observed, which is caused by the presence of the DIC2 molecule. Upon closer inspection of all the compared SA-DIC crystal structures, several detailed changes could be observed. Generally, there are not too many large changes in the conformation of amino acid side chains inside the binding pockets. In the DIC2 binding pocket in the CSA-DIC complex, the change of Met547 side chain conformation increases the space where a ligand is bound. In the FA6 pocket, the side chains of Arg208, Lys211, and Lys350 move to create interactions with the ligand, both in OSA and CSA. All changes in the Ib domain in the OSA-DIC complex are primarily caused by a large movement of the Trp134 side chain, which caused conformational changes of Tyr160, Asn161, and Met184.

### 2.1. Common Diclofenac Binding Sites in OSA-DIC, CSA-DIC, and LSA-DIC 

**Figure 1:**
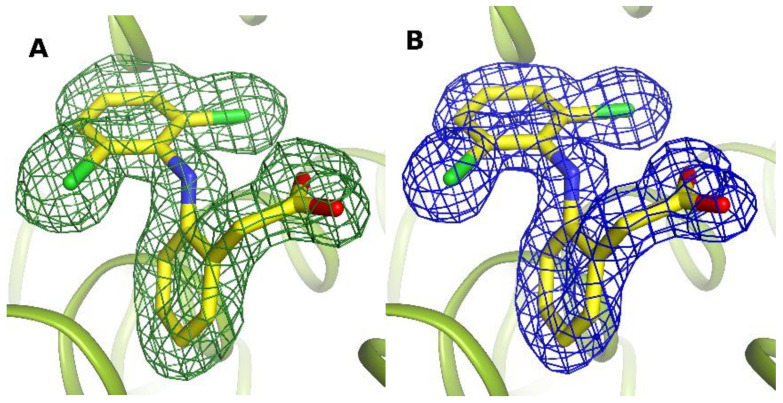
Electron density maps for DIC1 molecule in caprine serum albumin: *Fo-Fc* omit map contoured at 2.5 σ (**A**), and *2Fo-Fc* electron density map contoured at 1.2 σ (**B**).

A diclofenac molecule can interact with amino acids in several different ways: by creating hydrogen bonds, electrostatic interactions including halogen bonds or polar interactions, as well as hydrophobic contacts [32,33]. All identified diclofenac binding sites were clearly visible in electron density maps (Figure 1).

**Figure 2:**
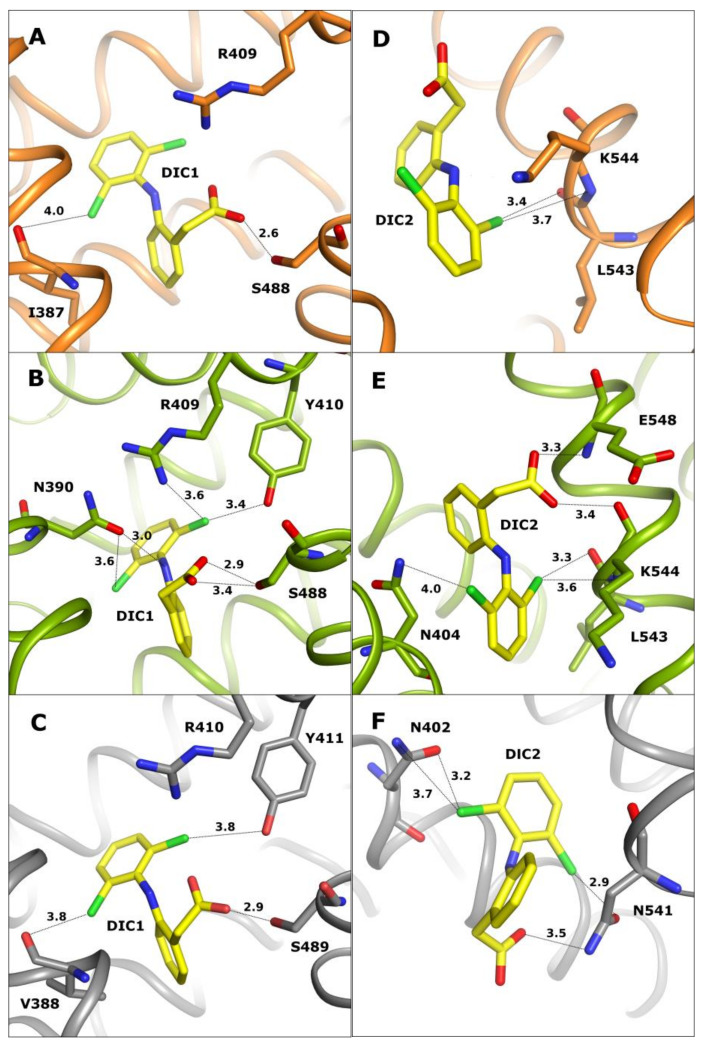
Interactions of diclofenac in DIC1 and DIC2 binding sites. Diclofenac DIC1 bound in drug site II in OSA (orange) (**A**), CSA (green) (**B**), and LSA (grey) (**C**). Diclofenac DIC2 bound in a cleft in the III domain (entrance to the FA5) in OSA (**D**), CSA (**E**), and LSA (**F**) (colored as in **A**–**C**, respectively).

The first common diclofenac binding pocket (DIC1) is localized in drug site II (DSII) (Figure 2A–C) in the IIIa domain, where a number of drug molecules are usually bound in serum albumins. 

This pocket is closed and surrounded by helices, and a number of polar residues create contacts with the ligand. In each discussed complex, the DIC1 molecule has an identical conformation and occupies the same space in the pocket. Even if there are several differences in the amino acid sequence, they do not influence the ligand binding. Diclofenac creates numerous hydrogen and electrostatic contacts with amino acid residues: Arg409 in OSA and CSA (Arg410 in LSA), and Tyr410 and Ser488 in CSA (Tyr411 and Ser489 in LSA) (Figure 2A–C). In the OSA-DIC complex (Figure 2A), the carboxyl group of diclofenac interacts directly with the hydroxyl group of Ser488. The chlorine atoms Cl4 and Cl2 connected to the DIC aromatic ring create electrostatic contacts with NH2 nitrogen of Arg409 and with the main chain oxygen of Ile387.

In the LSA-DIC complex (Figure 2C), the carboxyl group of diclofenac creates the same interaction with Ser489 as present in OSA-DIC. The chlorine atom Cl2 of this diclofenac molecule interacts with main chain oxygen of Val388 instead of interacting with Ile, the second chlorine atom interacts with Arg410 and Tyr411. It is noticeable that residues that are involved in ligand binding are very similar as for OSA. Some observed differences are probably caused by different contents of amino acid residues in the peptide chain (Lys389 into Gln390, Ile387 into Val388, or Gln384 into Lys385). The ligand molecule in the CSA-DIC (Figure 2B) complex creates the highest number of interactions. It is slightly shifted towards Ser488, making a polar interaction between its carboxyl group and hydroxyl group of Ser488 possible. As a result of its deeper location in this site, the ligand interacts almost with all the side chains of polar residues that are present in the pocket, but it loses the Cl2 chlorine contact with the main chain of Ile387. All contacts that are created by the carboxyl group and Cl4 chlorine atom in OSA-DIC and LSA-DIC complexes are also present here, and an additional interaction with the hydroxyl group of Ser488 is visible. In addition, the side chain carbonyl oxygen of Asn390 creates a halogen bond with Cl4 of DIC1 and additionally interacts via a weak hydrogen bond with the DIC1 NH group, acting as a hydrogen bond donor for the carbonyl oxygen of Asn390.

The second most common diclofenac binding site (DIC2) (Figure 2D–F) is localized in the narrow and elongated cleft between domains IIIa and IIIb in the entrance to the FA5 pocket (according to FA numbering in HSA structures: PDB IDs: 1E7E and 1E7G [5]). Different ligands are bound along this pocket in such a way that they interact with amino acids from one side and they are exposed to the solution from the other side. The shape and size of this pocket enables binding of diclofenac in different orientations, but DIC2 is bound in OSA and CSA in the same place in this pocket. In the CSA-DIC structure, the carboxyl group of DIC2 is rotated toward the side chain of E548 and makes additional interactions with the oxygen atom of peptide bond of K544 and nitrogen atom of E548. Some electrostatic interactions are common for the DIC molecule in these complexes, such as between chlorine Cl2 and peptide bond atoms: carbonyl oxygen of Leu543 and nitrogen of Lys544. 

In OSA-DIC, the ligand Cl4 chlorine atom interacts with its own carboxyl group (Figure 2D) and additionally creates weak halogen bonds with ND1 of His397, and carbonyl oxygen of Gly401. The aromatic ring of the benzoyl moiety is stabilized by hydrophobic interactions with the Met547 side chain. The other conformation of the DIC2 carboxyl group in the CSA-DIC structure (Figure 2E) enables it to make contacts with the main chain atoms of residues E548 and K544. Its chlorine Cl4 atom creates an electrostatic interaction with the ND nitrogen of Asn404. In the LSA-DIC complex (Figure 2F), the ligand molecule is shifted along the pocket and rotated by 180 degrees due to two sequence differences of amino acids. The helix 540–550 in LSA is shifted in the C-direction almost by one turn in comparison to CSA and OSA, and tilted from Val548 towards two other helices of the IIIB domain. Additionally, Leu544 and Lys545 reduce the depth of this pocket, which partially occupies the space where the aromatic ring of diclofenac is present in the OSA-DIC and CSA-DIC complexes. This conformational change causes a rotation of the DIC2 molecule and modifies its interactions in this binding pocket, but the location of the aromatic ring functionalized by chlorine atoms stays almost in the same place due to the interaction via a halogen bond that is created between Cl2 and the amide group of Asn402 side chain. A second halogen bond is present between the Cl4 chlorine atom and oxygen OD1 of Asn541 side chain. Additionally, the hydrogen interaction between DIC2 carboxyl group and amide nitrogen ND2 of Asn541 strongly anchors the ligand molecule inside this pocket. The orientation of the DIC2 molecule in LSA is very similar to the ESA-DIC complex [27].

**Figure 3:**
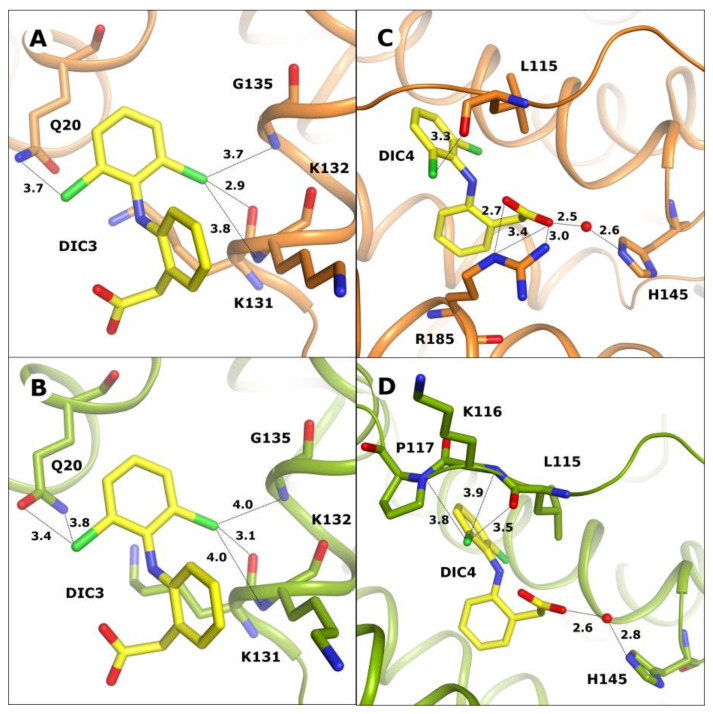
DIC3 binding site in albumins on I domain surface: in OSA (**A**) and CSA (**B**). DIC4 localized in the FA1 pocket: in OSA (**C**) and in CSA (**D**).

The third common diclofenac binding site (DIC3) of OSA and CSA is located on the surface of the first domain (Figure 3A,B) in a shallow, open cavity between its two subdomains. In this binding site, one aromatic ring of diclofenac is localized inside the pocket, and the other one, with the attached carboxyl group, is on the protein surface where it is partially surrounded by amino acid side chains. The DIC3 molecule is present in this binding site in OSA-DIC and CSA-DIC complexes, but in LSA-DIC, this pocket is ligand-free, probably due to several sequence differences including amino acids taking part in ligand binding, as well as some side chains changing the shape and surface charge of the pocket. In OSA and CSA, the tryptophan residue (Trp134) that is characteristic of even-toed ungulates’ serum albumins is localized in this pocket. There are no sequence differences between OSA and CSA in this pocket, only small dissimilarities in side chain conformation. As a result, the bound diclofenac molecule has an identical position and conformation in both complexes and nearly identical interactions are created (Figure 3A,B). The Cl4 chlorine atom in OSA-DIC interacts with NE2 nitrogen of Gln20. In CSA-DIC, chlorine Cl4 interacts with OE1 oxygen of Gln20. The second chlorine atom creates electrostatic interactions with the atoms of the main chain peptide bond: carbonyl oxygen of Lys131, and nitrogen atoms of Lys132 and Gly135.

**Figure 4:**
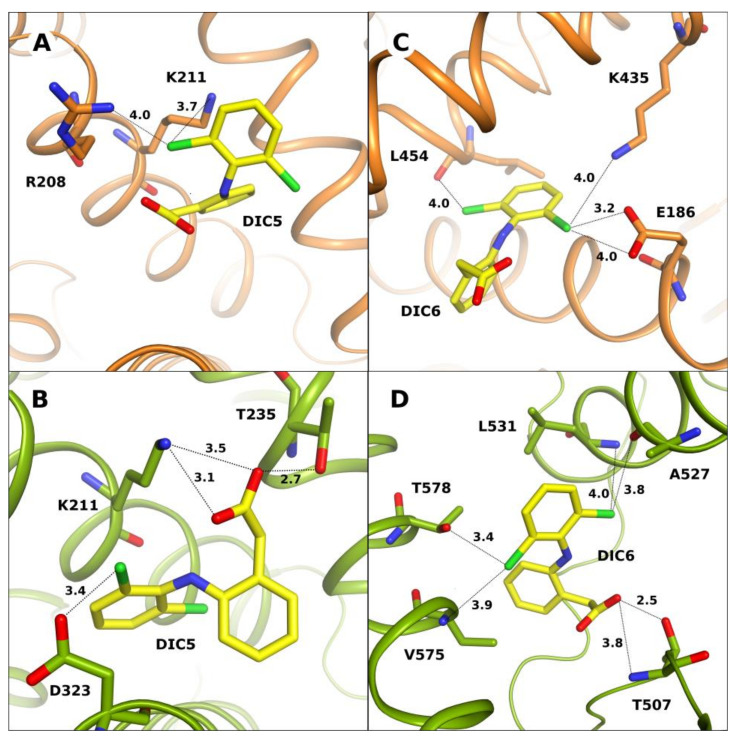
DIC5 binding site in the FA6 pocket, in OSA (**A**) and in CSA (**B**). DIC6 unique binding places: in the cleft between I and III domain, in OSA (**C**) and in IIIb subdomain, in CSA (**D**).

The fourth diclofenac (DIC4) binding site is observed only in OSA-DIC and CSA-DIC complexes (Figure 4C,D). The ligand is bound in the fatty acid binding site 1 (FA1) in a spacious and loose pocket near the protein surface, surrounded by three parallel helices and one flexible loop. There are no sequence differences influencing the composition of amino acid side chains in this pocket, only small dissimilarities in conformation are present. As a result, the ligand has an identical conformation in both discussed complexes and similar interactions are observed. The hydrogen bonds that are created with the participation of water molecules play an important role due to the large volume of this pocket. The diclofenac molecule in the OSA-DIC complex (Figure 3C) creates electrostatic contacts with its chlorine Cl2 atom and the peptide bond carbonyl oxygen of Leu115. The carboxyl group of this ligand creates a hydrogen bond through a water molecule with the NE2 nitrogen of His145 and the guanidine moiety of Arg185.

In the CSA-DIC complex (Figure 3D), the ligand creates almost the same interactions, with the exception of the electrostatic contacts with Arg185 which are lost due to a change in its side chain conformation. Instead of that, contacts with the main chain nitrogen of Lys116 and the main chain peptide carbonyl oxygen of Pro117 are observed. In the LSA molecule, there is a significant sequence inconsistency with OSA and CSA in the area of this pocket. The flexible loop forming FA1 is the place of additional amino acid insertion in the LSA sequence that causes a loop dislocation, and together with an alteration of several amino acids, changes the architecture of the binding site. In LSA instead of diclofenac, this pocket bound a poly-propylene glycol molecule that is present in the crystallization solution.

The DIC5 molecule in OSA-DIC and CSA-DIC complexes occupies the same part of the FA6 pocket, but has a significantly different conformation, it is rotated by 90 degrees around the NH moiety that is placed between two of the ligand’s aromatic rings. This spacious pocket is located on the surface of the protein molecule and is partially open to the solution space. The ligand molecule that is bound here interacts mainly with polar amino acid side chains (Figure 4A,B). In CSA-DIC, the ligand creates more electrostatic interactions than in OSA-DIC. The diclofenac carboxyl group creates hydrogen bonds with the NZ nitrogen atom of Lys211 and the hydroxyl oxygen of Thr235. Its chlorine atom creates an electrostatic interaction with the OD oxygen of Asp323. In the OSA-DIC complex, the carboxyl group of DIC5 does not create any strong contacts, only the chlorine atoms interact with the NZ nitrogen of Lys211 by electrostatic forces.

### 2.2. Unique Diclofenac Binding Sites in OSA-DIC and CSA-DIC 

In crystal structures of OSA and CSA complexes with diclofenac, unique binding sites for this ligand are observed. These sites are empty in other discussed albumins, including complexes that are deposited earlier in the PDB. This fact probably indicates lower ligand affinity in these pockets, which could be not occupied in physiological bloodstream conditions or its presence may depend on drug concentration. A high concentration of the ligand in the soaking procedure allows for mapping of all the binding sites, including those with weaker affinity.

In the CSA-DIC complex, the DIC6 molecule is localized in a unique place, inside the IIIb subdomain, covering the FA5 binding pocket (Figure 4D). Diclofenac is bound in a loose and partially open pocket. Hydrophobic interactions of diclofenac aromatic rings are observed here, as well as polar ones. The carboxyl group of DIC6 creates hydrogen bonds with the main chain nitrogen and OG1 oxygen of Thr507. Its chlorine atoms interact with the main chain oxygen of Ala527, nitrogen atoms of Leu531 and Val575, and side chain OG1 of Thr578. This pocket is empty in the crystal structure of the OSA-DIC complex.

In OSA-DIC, a unique diclofenac binding site for DIC6 is observed in the large space between the first and the third domains, located above the place where these domains are approaching each other (Figure 4C). This pocket is composed of many polar residues that are capable of interacting with the ligand. The aromatic ring functionalized with chlorine atoms acts as an anchor, creating electrostatic interactions in the deeper part of this cleft. The Cl4 chlorine atom interacts with the main chain peptide oxygen of Leu454 and Cl2 interacts via two halogen bonds with the carbonyl oxygen of Glu186 and the side chain amine group of Lys435. The chlorine atoms of DIC6 create more electrostatic interactions with polar residues than in the other pockets, and additional hydrophobic contacts cause its very strong stabilization in this pocket. The second aromatic ring occupies the wider part of the groove and creates hydrophobic contact with Leu189, and π-stacking with Arg458, but its methylene carboxyl group interacts only with the solvent.

### 2.3. Comparison of Diclofenac Binding Sites in All Known Structures of SA-DIC

A comparison of the diclofenac binding sites in serum albumins is possible based on the three crystal structures that were investigated in this work OSA-DIC, CSA-DIC, LSA-DIC, and two crystal structures that were determined earlier: ESA [27] and HSA [28] (Table 1).

These structures allow us to identify the most specific DIC binding sites in different serum albumins. These studies can help to understand exactly what influences the differences in ligand binding mode between serum albumins of different species.

Equine serum albumin (ESA) binds diclofenac (PDB ID: 4ZBQ) in two places which are also occupied in OSA, CSA, and LSA. The first of them is a common drug site II (DSII) and the second is inside the third domain. In the first binding pocket, DIC1 is bound in ESA in the same way as in other albumins. Almost the same interactions are observed, even if slight sequence differences are noticeable. These differences do not affect any hydrophilic residues of the pocket that participate in interactions with the ligand. This binding site could be identified as the primary binding pocket for diclofenac due to its presence in the majority of the discussed structures and similarity of the created interactions, which are the result of sequence conservation for active residues. The other diclofenac binding site in ESA-DIC is sequentially more similar to LSA than OSA or CSA, therefore, the ligand has a similar conformation to that in the LSA-DIC complex. However, the aromatic ring with the carboxyl group is rotated 90 degrees, causing different hydrophobic interactions due to different amino acid residues that are present in both proteins (Asp540 in ESA and Lys540 in LSA). 

HSA-PA-DIC has three diclofenac binding sites, but only one of them, located in the spacious FA1 pocket, is near the DIC4 molecule bound in OSA and CSA. The HSA-PA-DIC complex was crystallized in the presence of a fatty acid, and this hydrophobic molecule pushed diclofenac to a deep corner of this pocket. The space in this pocket has an ability to enlarge due to the movement of the loop covering this region. Its versatile binding properties allow it to bind various large ligands.

**Figure 5:**
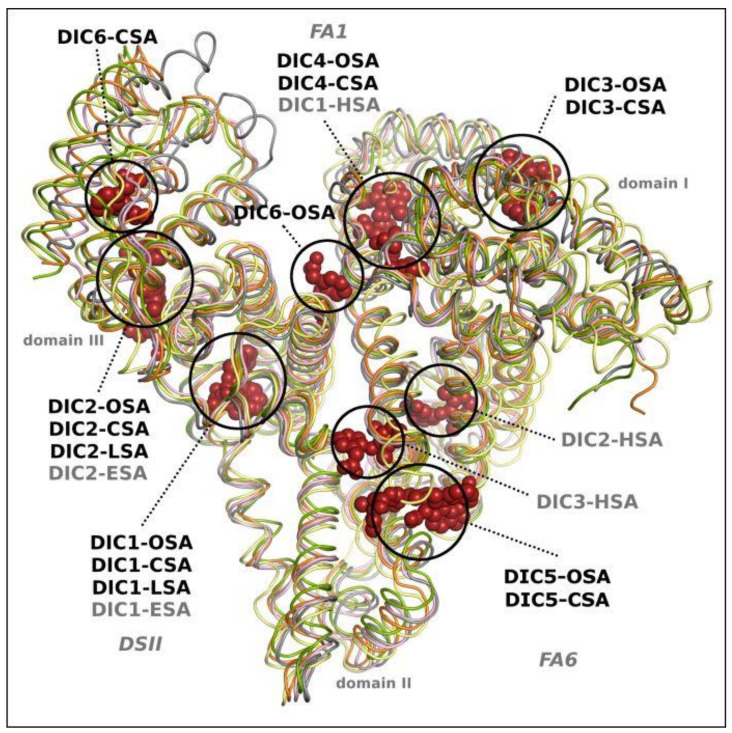
The diclofenac binding sites (DIC) in serum albumin crystal structures: OSA-DIC (ovine) CSA-DIC (caprine), LSA-DIC (leporine), ESA-DIC (equine) [27], and HSA-PA-DIC (human) (chain A) [28].

Structural alignment of the investigated serum albumin complexes with diclofenac that are presented in this work (OSA-DIC, CSA-DIC, LSA-DIC), and the previously studied ESA-DIC [27], allows us to compare the ligand location with the binding places in human serum albumin (HSA-PA-DIC) [28], which was saturated with palmitic acid (PA) before crystallization. LSA and ESA bind two molecules of DIC, OSA and CSA bound six ligand molecules, whereas HSA in complex with DIC and PA binds three diclofenac molecules in chain A and one in chain B (Figure 5).

There are two diclofenac binding sites that are identical for the discussed SAs, excluding HSA; in drug site II (DSII) and in domain III, DIC1 and DIC2, respectively. The other three molecules of diclofenac (DIC3, DIC4 and DIC5) bind at the same place in OSA and CSA, in domain III, FA1 and FA6 binding sites, respectively. In the FA1 pocket, the DIC1 molecule is present in the HSA-PA-DIC complex, in the deeper part of this pocket. In the place that is occupied by DIC4 in OSA and CSA, the palmitic acid is bound in the HSA complex. The last diclofenac (DIC6) binding site in OSA and CSA occupies a different pocket, characteristic only for each of these albumins.

## 3. Materials and Methods

### 3.1. Protein Purification, Crystallization, and Diffraction Measurement

Ovine (OSA), caprine (CSA), and leporine (LSA) serum albumins (Sigma Aldrich, St Louis, MO, USA and Equitech-Bio Inc., Kerrville, TX, USA) were purified using a previously described two step method [34]. In the first step, proteins were purified on activated carbon to remove any fatty acids or other possible contaminants. The second step was gel filtration on the fast protein liquid chromatography (FPLC) system (Amersham Biosciences, Uppsala, Sweden) to exclude albumin dimers that were present in the protein sample. Proteins were concentrated using Vi-vaspin filters (Sartorius, Göttingen, Germany) and centrifuged (Eppendorf, Hamburg, Germany). The final concentration of albumins was measured on a NanoDrop UV-VIS Spectrophotometer (Thermo Scientific, Wilmington, NC, USA).

All albumins were crystallized in their native form by vapor diffusion method and the hanging drop technique, with unique crystallization conditions for each protein. Ovine serum albumin was concentrated to 50 mg/mL and crystallized in the form of needles in the presence of an organic salts mixture–80% Tacsimate, pH 7.3, additionally, PEG400 was added to the protein solution at a final concentration of 2% to improve the crystal morphology. Caprine serum albumin crystallized as rhomboid prism crystals in the presence of 30% Jeffamine ED-2001, pH 7.0, 0.1 M sodium citrate buffer, pH 5.0 and 0.01 M barium chloride used as an additive to the crystallization drop, with a protein concentration of 120 mg/mL. Leporine serum albumin gave orthorhombic crystals from solution containing: 16% PEG3350, 8% PPG400, 0.2 M ammonium acetate, and 0.1 M TRIS, pH 8.0. Crystallization plates, crystallization screens, Tacsimate™ pH 7.0, and Jeffamine ED-2001 were purchased from Hampton Research (Hampton Research, Aliso Viejo, CA, USA). Other reagents were purchased from Sigma Aldrich (Sigma Aldrich, St Louis, MO, USA).

To obtain protein complexes with diclofenac (Sigma Aldrich, St Louis, MO, USA), the drug in the form of powder was added to the mother liquors with native crystals and the mixtures were incubated for 1–5 h in the case of OSA and CSA, and for 5 days with LSA. Crystals were flash-cooled in liquid nitrogen, and diffraction data were collected at 100 K on the BL14.2 beam line of Bessy Synchrotron in Berlin, Germany (CSA-DIC and LSA-DIC) [35,36], and on the PX14 beam line of Petra III, EMBL in Hamburg, Germany (OSA-DIC) without additional cryoprotection, because the components of the crystallization solutions had sufficient cryoprotective properties [37,38].

### 3.2. Diffraction Data Processing and Crystal Structures Determination

Diffraction data processing was done in XDS; scaling and merging were performed in XSCALE [39,40] to a resolution of 2.30 Å for OSA, 1.55 Å for CSA, and 1.89 Å for LSA, in space groups P3_2_21, P2_1_2_1_2_1_ and P2_1_2_1_2_1_, respectively. All crystal structures were solved by molecular replacement using Molrep (version 11.2.08) [41] and Phaser (version 2.5.6) [42] with native protein crystal structures (PDB IDs: 4LUF, 5ORI, and 4F5V) as models [34,43]. They were refined with REFMAC5 (version 5.8.0103) [44] from the CCP4 package (6.5.001) [45], and models were rebuilt in COOT (version 0.8.1) [46,47]. Additionally, to confirm ligand localizations in the structures, omit maps were generated in Polder [48] from the phenix package. The quality of the final structures was checked with MolProbity (version 4.2) [49] on the PDB validation server (https://validate-rcsb-1.wwpdb.org/). The final models were deposited in PDB (https://www.rcsb.org/) with accession codes 6HN0, 6HN1, and 8BSG for OSA-DIC, CSA-DIC, and LSA-DIC, respectively. A summary of the diffraction data collection and the refinement statistics are shown in Table 2. All figures were prepared in PyMol (version 1.9.0).

## 4. Conclusions

In this study, we aimed to gain a structural insight into diclofenac binding by two ruminant and one leporine serum albumins, which enriches the knowledge about the interactions of this drug with mammalian SAs. Sequence differences that were created during the evolutionary process of animal albumins slightly influenced their overall folding; however, their binding properties are visibly varied. This new structural information is valuable for showing the differences in the properties of serum albumins when interacting with this common NSAID. The diclofenac molecule interacts with two binding sites in leporine (LSA) and equine (ESA) serum albumin, which are the same in ovine (OSA) and caprine (CSA) albumins. These two ruminant albumins bound four additional DIC molecules; the location of three of them is common for both complexes, but the last one (DIC6) is unique for these two very similar albumins.

The first DIC1 molecule, common for all discussed albumin complexes, is bound in the drug site II (DSII). In this highly evolutionary preserved pocket, small differences in amino acid sequence do not influence the ligand binding properties. All DIC molecules that are present here, have similar conformation and occupy the same space in the pocket. It allows one to presume that DSII acts as the primary binding site for DIC and it has the highest specificity for this drug. The DIC2 molecule is localized in the narrow and elongated cleft between domains IIIa and IIIb. In OSA-DIC and CSA-DIC, the ligand occupies the same space in the pocket and its orientation is almost the same, with only the carboxyl groups bent in the opposite directions. In the LSA-DIC complex, changes in amino acid compositions cause a shift of the DIC2 molecule along the pocket and its rotation by 180 degrees. However, the localization of the aromatic ring functionalized by chlorine atoms remains almost the same due to the strength of the interactions. These two preferable binding sites not only display the highest affinity for diclofenac but also show high stereoselectivity for chiral profens [50].

There are also three diclofenac binding sites (DIC3-DIC5) that are common for OSA and CSA. DIC3 is localized in a shallow hydrophobic open cavity between two subdomains of the first domain. In both complexes, the chlorinated aromatic ring of diclofenac is bound deeper inside the pocket, while the second one, with an attached methylene carboxyl group, slightly sticks out of the pocket and it is only partially surrounded by amino acid side chains that are present on the protein surface. The DIC4 molecule is present in both complexes in a spacious and loose fatty acid binding site 1 (FA1). The ligand molecule has an identical conformation in both structures and it creates similar interactions, including hydrogen bonds that are created with the side chains of surrounding amino acids via a water molecule.

The DIC5 molecules in OSA-DIC and CSA-DIC complexes are located at the entrance to the spacious FA6 pocket, which is localized on the surface of the protein molecule and is partially open to the solution space. The diclofenac molecules are bound in both albumins, in different conformations, but their methylene carboxyl groups are similarly exposed to the solvent. The DIC5 of caprine albumin is anchored deeper in the FA6 pocket, whereas in the ovine albumin it sticks outside. The DIC6 binding site in ovine and caprine albumins is different. In OSA, it is a cleft between domains I and III, whereas in CSA it is inside the IIIb subdomain. 

In summary, based on the determined crystal structures, we postulate that reliable results for showing a particular ligand binding site in the albumins should be obtained without the presence of fatty acids, as they influence the affinity to other ligands. The experimental crystal structures of albumins in complexes with drugs provide a solid foundation for further studies on their distribution in organisms.

## Figures and Tables

**Table 1:** Known crystal structures of serum albumins with diclofenac.

PDB IDs of SA Complexes with DIC	Serum Albumin	Number of DIC	References
6HN0	Ovine (OSA-DIC)	6	This work
6HN1	Caprine (CSA-DIC)	6	This work
8BSG	Leporine (LSA-DIC)	2	This work
4ZBQ	Equine (ESA-DIC)	2	[27]
4Z69	Human (HSS-PA-DIC)	Chains: A/3, B/1	[28]

**Table 2:** Diffraction data collection and refinement statistics.

Data Collection	OSA-DIC PDB ID: 6HN0	CSA-DIC PDB ID: 6HN1	LSA-DIC PDB ID: 8BSG
Space group	P3_2_21	P2_1_2_1_2_1_	P2_1_2_1_2_1_
Unit cell (Å, º)	a = 121.7, b = 121.7, c = 121.8 α = 90.0, β = 90.0, γ = 120.0	a = 42.0, b = 66.3 c = 212.6 α = 90.0, β = 90.0, γ = 90.0	a = 74.8, b = 80.1, c = 104.1 α = 90.0, β = 90.0, γ = 90.0
V_M_ [Å^3^/Da]	3.9	2.2	2.3
Radiation source	BX14 Petra	BESSY BL.14.2.	BESSY BL.14.2.
Resolution range (Å)	50–2.12 (2.22–2.12)	50–1.55 (1.65–1.55)	50–1.89 (1.99–1.89)
Wavelength [Å]	0.976	0.918	0.918
R_merge_ † (%)	13.0 (142.7)	4.9 (72.6)	6.0 (132.7)
Mosaicity (º)	0.064	0.328	0.430
Completeness (%)	98.9 (97.2)	97.9 (91.9)	99.7 (100)
Redundancy	9.1 (8.7)	5.9 (4.3)	7.18 (6.95)
I/σI	14.6 (2.1)	22.1 (2.1)	21.67 (2.09)
R_free_ reflections	2941	1111	2533
**Refinement**			
Molecules in asymmetric unit	1	1	1
R/Rfree ‡ (%)	17.3/21.0	16.6/23.9	19.8/25.2
Protein/ligand/water atoms	4669/185/375	4714/115/501	4669/107/385
R.m.s.d. bond lengths (Å)	0.019	0.022	0.020
R.m.s.d. bond angles (◦)	1.822	1.981	2.040
<B> protein (Å ^2^) overall	45.1	28.0	49.95
TLS bodies	13	-	13
Ramachandran plot			
Most favored regions (%)	97.7	97.2	95.65
Allowed regions (%)	2.3	2.8	4.35

Values in parentheses correspond to the last resolution shell. † R_merge_ = Σ_h_Σ_j_|I_hj_ − ⟨I_h_⟩|/Σ_h_Σ_j_I_hj_, where I_hj_ is the intensity of observation j of reflection h. ‡ R = Σ_h_||F_o_| − |F_c_||/Σ_h_|F_o_| for all reflections, where F_o_ and F_c_ are observed and calculated structure factors, respectively. R_free_ is calculated analogously for the test reflections, randomly selected, and excluded from the refinement.

## Data Availability

Not applicable.
